# Automated Synapse Detection Method for Cerebellar Connectomics

**DOI:** 10.3389/fnana.2022.760279

**Published:** 2022-03-11

**Authors:** Changjoo Park, Jawon Gim, Sungjin Lee, Kea Joo Lee, Jinseop S. Kim

**Affiliations:** ^1^Department of Biological Sciences, Sungkyunkwan University, Suwon-si, South Korea; ^2^Laboratory of Computational Neuroscience, Korea Brain Research Institute, Daegu, South Korea; ^3^Department of Brain and Cognitive Sciences, Daegu Gyeongbuk Institute of Science and Technology, Daegu, South Korea; ^4^Laboratory of Synaptic Circuit Plasticity in Neural Circuits Research Group, Korea Brain Research Institute, Daegu, South Korea; ^5^Department of Electrical Engineering and Computer Science, Daegu Gyeongbuk Institute of Science and Technology, Daegu, South Korea

**Keywords:** connectomics, cerebellum, synapse, electron microscopy, image analysis, machine learning, computer algorithm

## Abstract

The connectomic analyses of large-scale volumetric electron microscope (EM) images enable the discovery of hidden neural connectivity. While the technologies for neuronal reconstruction of EM images are under rapid progress, the technologies for synapse detection are lagging behind. Here, we propose a method that automatically detects the synapses in the 3D EM images, specifically for the mouse cerebellar molecular layer (CML). The method aims to accurately detect the synapses between the reconstructed neuronal fragments whose types can be identified. It extracts the contacts between the reconstructed neuronal fragments and classifies them as synaptic or non-synaptic with the help of type information and two deep learning artificial intelligences (AIs). The method can also assign the pre- and postsynaptic sides of a synapse and determine excitatory and inhibitory synapse types. The accuracy of this method is estimated to be 0.955 in F1-score for a test volume of CML containing 508 synapses. To demonstrate the usability, we measured the size and number of the synapses in the volume and investigated the subcellular connectivity between the CML neuronal fragments. The basic idea of the method to exploit tissue-specific properties can be extended to other brain regions.

## Introduction

Cajal’s neuron doctrine was proven correct by the experiments in the late 1950s to 1960s, which directly observed the synapses with EM (Gray, 1959; Colonnier, 1968). Thanks to the advancement in molecular biology and optics, various methods to observe the synapses such as genetic labeling or immunochemical staining in combination with high-resolution light microscopes (LMs) are widely used (Ippolito and Eroglu, 2010; del Valle Rodríguez et al., 2011). However, the resolution limit of LM and the type specificity of the molecular markers often restrict the use of these technologies. Especially for the connectomics, whose ambition is to map the complete wiring diagram of nervous systems, EM is, presently, the only available solution since all the neurons and synapses in a nerve tissue are homogeneously imaged in EM (Denk and Horstmann, 2004).

A connectome is hypothesized to be the physical substrate of any mental processes of a life (White et al., 1986; Abbott et al., 2020). The connectome of a nematode, *C. elegans*, still remains the only complete connectome (White et al., 1986). Recently, a fruit fly connectome has become within reach, since a complete fruit fly brain was imaged by EM and semi-automated volumetric reconstruction is being performed (Zheng et al., 2018; Dorkenwald et al., 2020). It is foreseen that a mouse connectome will be the next goal and will become available within next 10 years (Abbott et al., 2020).

For connectomics, image analysis technologies are crucial to reconstruct the neurons and to detect the synapses from the EM image data. Recent advancement in the neuron reconstruction technologies, which are based on the deep learning AI, has rendered automatic reconstruction with small human intervention (Januszewski et al., 2018; Lee et al., 2019). Similar computational technologies have been developed for synapse detection as well (see Table 1 for the references). The advancement of synapse detection technology is lagging behind compared to that of the neuron reconstruction technology chiefly because the study for synapse detection began later.

**TABLE 1:** Accuracy of various synapse detection methods (selected animals).

Publication	Resolution (nm)	Animal	Region	Test set size (μm^3^)	F1-score
Kreshuk et al., 2011	5 × 5 × 9	Rat	Somatosensory cortex	241	0.905
Becker et al., 2013	5 × 5 × 5	Rat	Cerebellum	66	0.941
Becker et al., 2013	5 × 5 × 5	Rat	Hippocampus	58	1
Becker et al., 2013	6.8 × 6.8 × 6.8	Rat	Somatosensory cortex	22	1
Kreshuk et al., 2014	4.5 × 4.5 × 45	Mouse	Visual cortex	970	0.904
Plaza et al., 2014	10 × 10 × 10	Drosophila	Optic lobe	27,000	0.785*
Roncal et al., 2015	6 × 6 × 30	Mouse	Somatosensory cortex	114	0.815*
Dorkenwald et al., 2017	10 × 10 × 30	Mouse	Striatum	19,200	0.854
Dorkenwald et al., 2017	9 × 9 × 20	Zebra finch	Area X	909,000	0.905
Dorkenwald et al., 2017	9 × 9 × 21	Zebrafish larval	Spinal cord	422,000	0.858
Staffler et al., 2017	11.2 × 11.2 × 28	Mouse	Somatosensory cortex	237	0.883
Heinrich et al., 2018	4 × 4 × 40	Drosophila	calyx	375	0.877
Huang et al., 2018	10 × 10 × 10	Drosophila	Optic lobe	27,000	0.83*
Xiao et al., 2018	2 × 2 × 50	Mouse	Cortex	2,325	0.892
Parag et al., 2018	6 × 6 × 29	Mouse	Somatosensory cortex	164	0.93*
Buhmann et al., 2021	4 × 4 × 40	Drosophila	calyx	Whole region	0.74
Buhmann et al., 2021	4 × 4 × 40	Drosophila	Lateral horn	Whole region	0.68
Buhmann et al., 2021	16 × 16 × 40	Mouse	Cerebellum	320	0.94
Park et al., 2022 (this work)	12 × 12 × 50	Mouse	Cerebellum	1,757	0.955

The accuracies of recent synapse detection methods are shown together with the types of the data and animal species. The * symbol indicates that the value is read from a graph.

We have a practical motivation to develop an automatic synapse detection method since we plan to connectomically study the cerebellum. Although the connectomics concerns the entire brain, the studies on the neural circuits do not necessarily require a complete connectome. The investigations into the microcircuits in “partial connectomes” from diverse brain regions and species yield crucial knowledge’s of the fundamental principles of neural connectivity and function (Takemura et al., 2013; Kim et al., 2014; Ohyama et al., 2015). For the cerebellum, the development of the climbing fiber in the infant mouse cerebellum, a new type of Purkinje cell layer interneuron, and the connectivity between granule cells and Purkinje cells have been studied by 3D EM image analyses (Wilson et al., 2019; Nguyen et al., 2021; Osorno et al., 2021). Nonetheless, the functional circuit mechanisms of the motor control and learning of the cerebellum are still largely in veil. In search of the clues, we plan to investigate a small EM image volume of the CML of a mouse taken by serial block-face scanning EM (SBEM).

**FIGURE 1:**
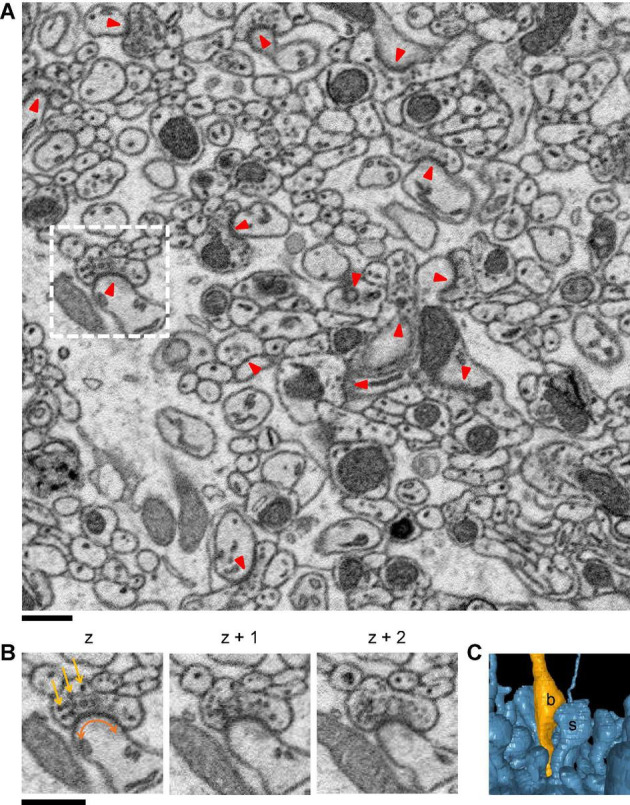
Image resolution is enough for visual identification of synapses and synaptic structures. **(A)** Various examples of synapses between different types of neuronal fragments are shown. They have presynaptic vesicles (small blobs) and postsynaptic density (a dark blurring or thick line on the membrane) regardless of the types. The red triangles point the synapses from the postsynaptic side. **(B)** About 3 consecutive sections of images for a synapse corresponding to the white dashed box. The yellow arrows point a few examples of neurotransmitter vesicles, and the curved brown arrow indicates the range of postsynaptic density. **(C)** 3D mesh rendering of the two neuronal fragments in the vicinity of the images in panel **(B)**. The label “b” denotes the bouton, and “s” denotes the spine. Scale bars: 600 nm, 50 voxels.

For semi-automated neuronal reconstruction, we employed one of the competitive technologies and a proofreading pipeline with paid workers (Kim et al., 2014; Lee et al., 2017). For synapse detection, we require a method that shows a practically applicable level of accuracy (>95%). Various synapse detection methods have been proposed, and their accuracy has increased during the last decade. However, only a few have been developed and tested specifically for the cerebellum, and none of them exceed the accuracy bound (Table 1). Therefore, we decided to develop a novel, fully automated method, which is specialized for the cerebellum and can handle our EM image data whose resolution (12 nm × 12 nm × 50 nm voxel size) is relatively low (Table 1 and Figure 1).

Such requirement is difficult to accomplish as seen from the preceding studies. In general, there are several challenges in the connectomic EM image analyses, for both reconstruction of neurons and synapse detection. First, the quality of EM images is not always ideal. The defects in the sample, which occur during tissue preparation or staining, hinder precise image analysis. The image resolution is often compromised over the expenses in time and money that large-scale imaging requires. Second, the neuronal structures have intrinsic biological ambiguity such as thin processes and small synaptic structures. Third, the image analysis technologies often generalize poorly, and they show good performance only for the data on which they are developed and tested. A new technology or extensive fine tuning is needed for new data.

To overcome such challenges, we developed a method that is specialized to the cerebellum because the goal seems to be unreachable with a general solution. It utilizes two deep learning AIs, prior knowledge of the cerebellum, and fine tuning of parameters. The method works for a 3D EM image volume, provided together with the reconstruction of the neuronal fragments and their type information. The contacts between a pair of neuronal fragments are extracted from the reconstruction. The contacts are classified into synaptic or non-synaptic through multiple steps; each of which selects a subset of the contacts from the previous step (Staffler et al., 2017). The first selection is conducted based on the types of the neuronal segments, and only the contacts between the types that can have synapses are chosen. The second and third selections are conducted with the aid of the AIs, which 3-dimensionally evaluate the visual cues of the synapses (Cicek et al., 2016; Lee et al., 2017). The method can also assign the synaptic partners into pre- and postsynaptic sides (Buhmann et al., 2021) and determine the excitatory and inhibitory types.

The method is shown suitable for the cerebellar connectomics research. It is applied to a small test volume to evaluate the accuracy and to showcase the usability. The accuracy is 0.955 in F1-score for the test dataset containing 508 synapses. The size and the density of the cerebellar synapses are inspected. The parallel fibers are shown to innervate the consecutive Purkinje cells along the transverse axis in a random manner. Although this method was designed for the cerebellum, the basic idea of specialization exploiting tissue-specific properties can be extended to other brain regions.

## Materials and Methods

### Electron Microscope Image and Reconstruction

An adult wild-type mouse was used, and a slice of cerebellar tissue was processed for SBEM following standard protocols (Briggman et al., 2011; Xu et al., 2020). The tissue was conventionally stained with the osmium compound and then infiltrated with epoxy resin. The specimen was cut and imaged roughly along the sagittal axis by a Merlin VP field emission scanning electron microscope (Carl Zeiss) equipped with 3View2 in-chamber ultramicrotome and a backscattered electron detector (Gatan). XY resolution was 12 nm, and nominal thickness was 50 nm. One block-face image was acquired in 2-by-3 tiles with 10∼20% of an overlap between the tiles, each of which is 5,000 by 5,000 pixels. Consecutive 1,000 block faces were imaged.

Each of the 6 stacks of 1,000 images was aligned separately and then merged using Image J and TrakEM2 plugin software (Cardona et al., 2012) together with in-house MATLAB codes. After the registration, the size of image volume is 14,600 × 10,200 × 1,000 voxels, approximately corresponding to a 175 μm × 122 μm × 50 μm physical dimension.

To automate the reconstruction of this large volume, an AI implemented by a modified 3D U-Net was employed (Cicek et al., 2016; Lee et al., 2017) to segment the images into different neurons using the cellular membrane as the boundary. To train the network, eight subvolumes from the entire volume were taken at various locations and sizes as training data. They were manually reconstructed by human experts (advanced paid workers) with specialized software, VAST (Berger et al., 2018). Then, the trained network segments the entire volume to reconstruct the putative neuronal fragments.

Since the AI-aided segmentation contains errors, it was proofread by paid workers to yield the final reconstruction using in-house software with an interactive graphical user interface and a few kinds of background software for work process management (Kim et al., 2014). The proofreading was conducted progressively for one neuronal fragment after another, and each neuronal fragment was represented by one segment after proofreading was done.

The segments of proofread neuronal fragments were saved in a separate volume. The separate volume gradually turns from sparse to dense as the proofreading progresses. We used a snapshot of such volume from a fixed date where 57.6% of the volume is filled with the segments of proofread neuronal fragments. This volume will be called as “completed segmentation” hereafter.

Further details of the animal, sample preparation, SBEM acquisition, alignment, image segmentation, and proofreading for 3D reconstruction will be reported elsewhere.

### Synaptic Structures

The mammalian synapses in EM images are characterized by the cloud of presynaptic neurotransmitter vesicles and the postsynaptic density (PSD), which are protein complexes specialized for synaptic transmission (Ziff, 1997). These structures are electron dense and visually prominent as they appear dark in EM images (Figures 1A,B and Supplementary Figure 1). Narrow synaptic clefts are also visible in high-resolution EM micrographs, but they are hardly observable when the image resolution is worse than roughly 10 nm per voxel, which is within the resolution range commonly used for connectomics (Table 1). In the 3D representation of reconstructed neurons, the presynaptic boutons (“b”), which appear as swelling on the axon and the postsynaptic spines (“s”), which appear as short protrusion from the dendritic shaft, are also characteristic (Figure 1C). Most of the synapse detection methods for EM, including human visual inspection and our method, take advantage of these visual cues.

### Overview of the Method

**FIGURE 2:**
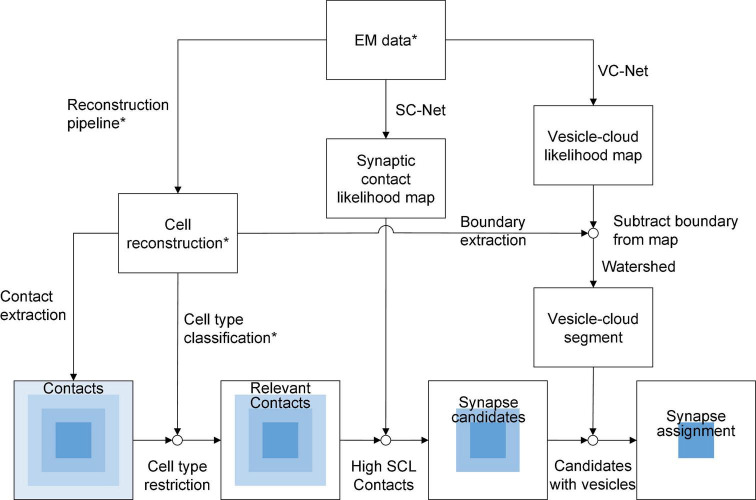
A procedure overview of the method. The diagram showing the overall procedures of the proposed synapse detection method. The * symbols indicate the prerequisite or input data to the method. From the reconstructed neuronal fragments, the contacts between neurons are extracted. The goal of the procedures is to filter these contacts and select only the synaptic contacts (bottom row). First, the contacts are restricted by the cell types to select the relevant contacts. Second, synapse candidate contacts are selected, whose voxels have high SC likelihood values evaluated by an AI. Finally, those contacts that have a VC in a close distance are determined as the synapses, where the VC is found by another AI. At the final step, the excitatory and inhibitory synapse types and the pre- and postsynaptic neurons are assigned.

The method assumes the EM image volume, the corresponding segmentation of reconstruction, and the type information of each neuronal fragment as the input (Figure 2). The contacts between pairs of neuronal fragments are extracted from the reconstruction. They are classified into synaptic or non-synaptic through 3 steps (Figure 2, bottom row).

**FIGURE 3:**
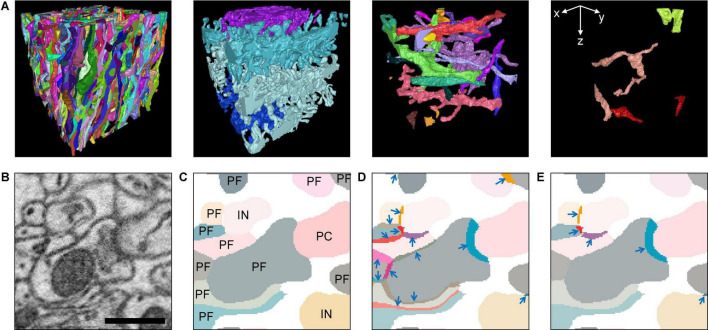
From cell reconstruction to relevant contacts. **(A)** The 3D renderings of the neuronal fragments in the test set are shown in the four panels grouped by the types. From the left: the parallel fibers (PF), Purkinje cells (PC), inhibitory interneurons (IN), and climbing fibers (CF). **(B)** A small example patch of EM image. **(C)** The reconstruction of B that shows the segments of neuronal fragments. The segments are color coded, and their types are shown. White background represents the space without reconstruction (57.6% of the imaged volume is reconstructed.) **(D)** The color-coded 3D contacts are overlaid on top of the segments of neuronal fragments. The arrows are pointing each contact separately. A contact is the union of the surface voxels from two segments of neuronal fragments that touch each other. Note that some contacts appear thicker than 2 voxels because the contacting voxels to the Z direction are also shown in the XY plane. **(E)** The relevant contacts are chosen between PF-PC and PF-IN among all the contacts in panel **(D)**. The PF-PF contacts were discarded because they could not make a synapse and were irrelevant. The type information of the neuronal fragments that form the contacts is kept until the contact is determined as synapse. The synapse types are determined based on the types of neuronal fragments. For example, all the relevant contacts in panel **(E)** are potentially excitatory synapses because they are PF-PC or PF-IN contacts. A scale bar: 600 nm, 50 voxels.

The first step selects the synaptically “relevant contacts” utilizing the type information. The cerebellar cortex has only a few anatomically distinct types of neuronal fragments, and the connectivity between the types is assumed to be regular. A type of neuronal fragments can have connections only to limited types of partners. A contact is regarded as relevant when it is made between such types (Figure 3). Since the neuronal fragments that make irrelevant contacts are known not to have any connection to each other, the irrelevant contacts can be excluded from the candidates for synaptic contacts. This efficiently reduces search space and helps decrease false positive errors, which is otherwise difficult (see section “Accuracy Evaluation”).

For the second and third steps, two 3D U-Nets are separately trained to evaluate the likelihood of each voxel being a synaptic contact (SC) voxel and a vesicle-cloud (VC) voxel based on the visual cues for synapses. They are called as SC-Net and VC-Net, respectively (Supplementary Figure 2). The networks learn from the examples in the training data where the SC voxels and VC voxels are annotated by human experts. The networks mimic what humans do and label the SC and VC voxels with a confident level, or the likelihood (Supplementary Figure 3).

**FIGURE 4:**
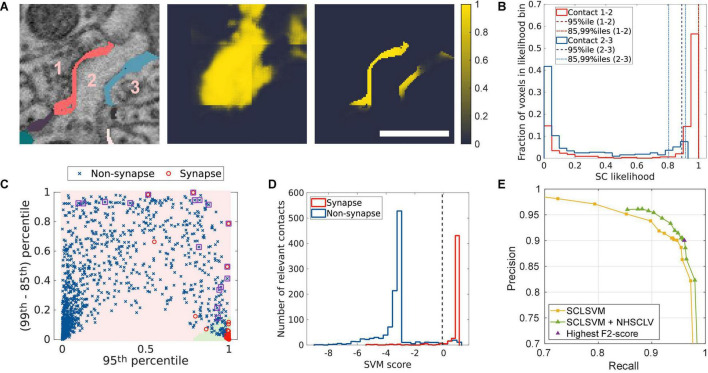
Synapse candidates are the contacts with high SC likelihood. **(A)** Two contacts are shown (first panel), one between Neurons 1 and 2 (red); one between Neurons 2 and 3 (blue). The SC-Net produces the SC-likelihood map of all the voxels (middle panel). The likelihood values only for the voxels belonging to each contact are collected (right panel) using the contacts as a mask. A scale bar: 600 nm, 50 voxels. **(B)** The distributions of the SC-likelihood values for the two contacts are shown together with 3 percentiles. The contact that is likely to be synaptic (1–2) has narrowly peaked distribution toward high values (red histogram). The 95th percentile (a red dash-dotted line) assesses the bias toward high values, and the (85th–99th) percentile range (a red dashed line) assesses the width of the peak. **(C)** When the two percentile measures are scatter plotted, synaptic contacts (a red circle) and non-synaptic contacts (a blue cross) from the training sets loosely segregate with a fuzzy boundary. A support vector machine (SVM; SC-likelihood SVM; SCLSVM) was trained to draw a decision boundary. The two regions separated by the boundary are colored with green (positive) and pink (negative) backgrounds, respectively. The contacts that have 400 or more voxels with 0.9 or higher SC likelihood are also considered as synapse candidates (number of high-SC-likelihood voxels; NHSCLV). The purple squares indicate the contacts that transit from negative to positive by this. **(D)** The histograms of the SVM scores of the synapses and non-synapses in the test set are shown. The dashed vertical line represents the usual decision boundary, a zero-SVM score. However, we wanted to accept a few false positives near the boundary to enlarge the pool of synapse candidates. **(E)** The PR curves for varying SVM thresholds are plotted, for the cases when only the SCLSVM is used and when both the SCLSVM and NHSCLV are used (see section “Finding Synapse Candidates Using SC Likelihood”). The F2-score, which measures the false-positive errors permissively, is the highest when the SVM threshold is –0.2 (a purple triangle). The SVM threshold for synapse candidate selection is decided to be –0.2.

**FIGURE 5:**
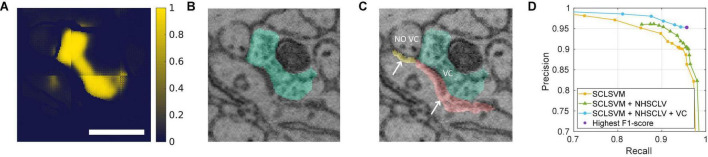
A synapse has a vesicle cloud in the presynaptic side. **(A)** The VC-Net produces the map of VC likelihood of all the voxels. The map is masked by the neuronal boundaries to prevent the VC from crossing multiple segments of neuronal fragments. A scale bar: 600 nm, 50 voxels. **(B)** The 3D VC segment (green) is obtained from the map by a watershed algorithm. **(C)** Two synapse candidate contacts are shown in yellow and red. For each synapse candidate contact, a VC segment is searched for. The yellow contact, which does not have a VC in any of the contacting neurons, is classified as non-synaptic. The red contact, which has a VC in one of the contacting neurons within 5-voxel distance, is classified as synaptic. **(D)** The PR curve when the VC requirement is added to the SCLSVM and NHSCLV, for varying VC size thresholds. The other two PR curves are repeated from Figure 4D for references. The final step gives the highest F1-score of 0.955, and the VC size threshold is decided to be 1,000 voxels.

In the second step, “synapse candidate” contacts are selected from the relevant contacts. The synapse candidates are those contacts whose voxels have an SC-likelihood distribution that has a peak at a high value (Figure 4). The final step determines the candidates as the synaptic contacts if a candidate contact has a VC in a close distance. The VC is a segment of connected voxels with high VC likelihood (Figure 5).

The assignment of the pre- and postsynaptic neuronal fragments is straightforward from the final step, because those containing the VC can be assigned as presynaptic and the other as postsynaptic. One presynaptic bouton can innervate multiple postsynaptic spines (Toni et al., 1999; Federmeier et al., 2002). The excitatory and inhibitory synapse types are determined based on the type information that is associated with the neuronal fragments that form each contact. The synapse type is determined following the excitatory vs. inhibitory nature of the presynaptic side.

### The Datasets

Seven subvolumes that were taken at various locations from the entire imaged volume were used for this work (Table 2). Each subvolume is the combination of an EM image volume and the corresponding segmentation volume (Figures 3B,C). Six out of the eight subvolumes, which were used for the training of segmentation AI, were used again for the training of the synapse detection AIs. The segmentations of these subvolumes were completely reconstructed by manual tracing as mentioned above (see section “Electron Microscope Image and Reconstruction”). For the SC-Net, five were used as training sets and one as a validation set. For the VC-Net, four were used as training sets and one as a validation set. The dataset 3 was used for SC-Net validation, and the dataset 4 was used for VC-Net validation. One additional subvolume, which is much larger than the training and validation sets, was prepared as a test set. The segmentation volume of the test set was taken from the completed segmentation volume, in which 57.6% of the volume is reconstructed (Figure 3A) by the semi-automated reconstruction (see section “Electron Microscope Image and Reconstruction”). All of the datasets mostly consist of neuropil, and the soma is only minimally included.

**TABLE 2:** The basic statistics of the datasets.

Dataset	ID	Size (voxels)	Reconstructed neuronal fragments	Contacts	Relevant contacts	Vesicle clouds	Synapses
Train and Validation	1	256 × 256 × 64	80	340	N/A	14	13
	2	256 × 256 × 64	92	346	N/A	17	13
	3	384 × 384 × 96	217	996	N/A	49	43
	4	384 × 384 × 96	184	802	N/A	44	46
	7	384 × 384 × 96	217	1,078	N/A	39	32
	8	512 × 512 × 128	184	846	N/A	N/A	29
Test		992 × 992 × 248	598	5,857	1,973	N/A	508

*The size, numbers of the segments of neuronal fragments, contacts, relevant contacts, vesicle clouds, and ground truth synapses are shown for the datasets.*

*The Datasets 1, 2, 4, 7, and 8 were used as training sets, and Dataset 3 was used as a validation set for the SC-Net training.*

*The Datasets 1, 2, 3, and 7 were used as training sets, and Dataset 4 was used as a validation set for the VC-Net.*

*The fields for irrelevant data are marked as “not applicable (N/A)”.*

### Type Classification of Neuronal Fragments

The cerebellar molecular layer contains four major types of neurons or neural processes (Figure 3A): Purkinje cell (PC); climbing fiber (CF), which is the axonal projection from the inferior olivary nucleus neurons; parallel fiber (PF), which is a part of the axon of the cerebellar granule cell; and molecular layer inhibitory interneuron (IN). Since only a part of the neurons or neural processes is in the data, we referred to all these as neuronal fragments for simplicity. The 3D mesh rendering of each neuronal fragment was visually inspected by human experts upon the completion of the proofreading. Human experts can tell the types from the completed segmentation volume (14,600 × 10,200 × 1,000 voxels, 175 μm × 122 μm × 50 μm size) where the large-scale context of the neuronal structure is preserved. PCs have spiny dendrites, PFs are long and straight along the transverse axis, CFs arborize parallelly to PCs, and INs have dendrites that are confined within the CML. The type information determined from the completed segmentation volume was transferred to the neural segments in the subvolumes when applicable (Figures 3A,C).

### Contact Extraction

The computations in the “Materials and Methods” section below were performed by custom written MATLAB codes unless otherwise noted. The contacts between a pair of neuronal fragments are extracted from the segmentation volumes of all the datasets (Figure 3D). When there were background voxels due to extracellular space or annotation gaps between neuronal segments in the volume, the segments were dilated until they saturated the volume to ensure that neighboring segments touched each other.

The segmentation volumes are a 3D-labeled image stack, where each voxel is labeled by a numerical ID of a segment (Figure 3C). To extract the contacts from these data, we searched the voxel locations that the segment ID changes values into 6-neighborhood. As the calculation is symmetric, contact voxels are found on both sides of a pair of neuronal fragments, yielding two-voxel thickness. All the contacting voxels between a pair of neuronal segments were grouped by connected component analysis, and each connected component is considered as one contact (Figure 3D). The contacts with 200 or less voxels (roughly 0.03 μm^2^ or less) were considered as noise and were excluded.

### Cell Type Restriction for Relevant Contacts

Only five pairs among the four types of neuronal fragments of the CML are known to have connections, from CF to PC, PF to PC, PF to IN, IN to PC, and IN to IN (Eccles et al., 1967; Kim and Augustine, 2021). The contacts between these five pairs were accepted as the relevant contacts (Figure 3E). The relevant contacts are required during the prediction of the synapses but not during the training of the AIs. The SC-Net and VC-Net are trained only by the EM images with the ground truth labels (see sections “Ground Truth and Data Labeling,” “SC-Net Architecture and Training,” and “VC-Net Architecture and Training”). Therefore, the relevant contacts are not computed for the training and validation sets (Table 2).

### Ground Truth and Data Labeling

The synapse structures in the datasets were labeled by human experts. First, the synapses are searched for and identified in the datasets. To find the synapses, two experts visually inspected all the extracted contacts in the training and validation sets and voted for synaptic, non-synaptic, or ambiguous using the visual cues discussed above as the criteria (see section “Synaptic Structures”). For the disagreements, the two experts had a debate on them and then voted again. The tenacious disagreements in the second voting were labeled as ambiguous. For the test set, only the relevant contacts were inspected and then labeled with the same method. The ambiguous cases were not used as the ground truth for AI training and were not included in the accuracy evaluation.

Next, the synaptic structures are actually marked on the data. The extracted contacts, which were consented as synaptic, directly became the SC in most cases (Figure 4A, left panel and Supplementary Figure 3A, left panel). Occasionally, however, the area of the PSD, which is biologically relevant area for synapse, is smaller than the contact. When it is the case, human experts erased the part on the contact that lies outside the PSD in the training and validation sets. The sizes before and after erasion of each contact can be used to assess the overestimation of approximating the synapse size by the contact size. The fraction of the size change, (size_before_−size_after_)/size_after_, is measured for each contact (section “Synapse Size and Density”).

The human experts also searched for and labeled a VC for each SC. The label for a VC is a 3D area inside the perimeter formed by the outermost neurotransmitter vesicles (Supplementary Figure 3B, left panel). We used VAST for erasing of the SC and labeling of the VC. The VCs were not labeled for Dataset 8, since it was used only for SC-Net training (Table 2).

### Measurement of Accuracy

The accuracy was measured in a contact-wise manner by comparing the results of the method and the labels by human experts. The contacts in the validation sets and the test set were labeled as synaptic, non-synaptic, or ambiguous from the voting. The method classifies each contact into synaptic and non-synaptic, and it is compared to the ground truth labeling. The experiments were performed on the test set, and we calculated the precision and recall by counting the true-positive (TP), false-positive (FP), and false-negative (FN) cases.


$$\text{Precision}=\frac{TP}{TP+FP},\text{Recall}=\frac{TP}{TP+FN}$$

The ambiguous contacts were not included in the calculation of accuracy. Regardless of the prediction on the ambiguous contacts, they were not counted as any of the true positive, false positive, nor false negative.

The accuracy was measured in *F*_β_ -score. The *F*_β_ -score, defined as below, is the generalization of the *F1*-score. *F1*-score (*F*_β_ -score for β = 1) is the harmonic mean of precision and recall. *F*_β_ -score gives more weight on recall when β > 1 (penalize false-negative errors more) and gives more weight on precision when β < 1 (penalize false-positive errors more).


$$F_{\beta }=\frac{\left(1+\beta^{2}\right)\cdot \text{precision}\cdot \text{recall}}{\beta^{2\cdot }\text{precision}+\text{recall}}$$

The precision-recall (PR) curves are used to decide the optimal parameters and calculate the maximum possible *F*_β_ -scores. In a PR curve, the precision and recall values for a varying, controllable parameter are drawn in a connected scatter-plot.

### SC-Net Architecture and Training

Both the SC-Net and VC-Net were implemented and trained using Caffe (Jia et al., 2014) with its Python interface (Lee et al., 2017).

The SC-Net takes an image volume as the input and produces the likelihood map of the voxels being SC as the output. The network architecture was adopted from the 3D U-Net (Ronneberger et al., 2015), and a few details were modified (Supplementary Figure 2). The sizes of the first two kernels were decided in such a way that the anisotropic data (different resolutions in XY and Z) to the input layer become isotropic to the inputs of the next layers. The dropout layers were introduced to avoid overfitting caused by the lack of training data (Srivastava et al., 2014). The number of parameters in each layer was increased from those of the original 3D U-Net to increase the efficiency of the dropout.

The data augmentation was employed to enhance the training data. It was applied on-the-fly as follows. In each training iteration, 3D patches (12 × 44 × 44 voxels) were sampled, and one of the four augmentation operations (flipping, misaligning, gray scaling, and warping) was randomly conducted. Masking was used to handle the class imbalance between SC- and non-SC voxels. The class imbalance hinders the training because any prediction biased toward the majority class would result in high accuracy (Provost and Weiss, 2003). The labeled datasets are extremely imbalanced. For example, one of the training datasets contains 14,155,776 voxels, and only 73,190 voxels are SC (0.5%). Therefore, the SC-Net was trained only with the boundary voxels of reconstructed neurons, masking out all the rest voxels.

The SC-voxels and non-SC voxels on the neuronal boundary are the positive and negative training data, respectively. The loss function is the sigmoid cross-entropy error. The learning rate was initially set to 1 × 10^–5^ and then multiplied by 0.98 every 6,000 iterations. The training was terminated after 1 million iterations when the error reached an asymptote (Supplementary Figure 3A).

### Finding Synapse Candidates Using SC Likelihood

The SC-Net output is the map of voxel-wise likelihood of a voxel being on an SC. The contact-wise likelihood for being synaptic was estimated as follows. The SC-likelihood values (Figure 4A, middle panel) were masked by the relevant contact voxels (Figure 4A, left panel) to collect only the SC-likelihood values for each relevant contact (Figure 4A, right panel). Note that the non-contact voxels can have high SC likelihood because the SC-Net was trained only with the boundary voxels using the mask (Figure 4A, middle panel). The likelihood values for non-contact voxels, no matter they are high or low, are irrelevant.

The distribution of the SC likelihood of the contact voxels reveals the likelihood of the contact being synaptic (Figure 4B). The synaptic contacts would have the distribution that is narrowly peaked at a high likelihood value. To capture such a distribution pattern, we utilized two percentile features. The 95th percentile represents the bias of the distribution toward high values. The range between the 85th and 99th percentiles indicates the width of the peak. Indeed, the synaptic contacts in the training sets tend to exhibit a high 95th percentile and a small 85th- to 99th percentile range (Figure 4C). However, there is a gray zone in the plot, and the boundary between the synapses and non-synapses is ambiguous. A support vector machine (SVM) was recruited to decide the boundary. The three parameters for percentile (95; 85 and 99) were chosen from many experiments to yield the best accuracy (data not shown).

Conventionally, the decision threshold of an SVM is the zero SVM score. The result for the test set shows numbers of false-positive and false-negative errors when the zero threshold is used (Figure 4D). We wanted to prioritize making less false-negative errors to making less false-positive errors, because false-positive errors could be eliminated at later stages, but false-negative errors are lost once they are excluded from the candidates. To this end, the SVM threshold was tuned using the PR curve for the test data (Figure 4E). The SVM threshold −0.2, which gave the maximum F2-score, 0.947, was chosen.

The inspection on the errors revealed a special error mode, which is that the fraction of high SC-likelihood voxels is small because the synaptically relevant part of the contact is much smaller than the entire contact (see section “Ground Truth and Data Labeling”; Supplementary Figure 4). To rescue these errors, we added a voxel-count based rule (number of high-SC-likelihood voxels; NHSCLV), in addition to the voxel fraction-based SVM decision that a contact is synaptic if at least 400 contact voxels have 0.9 or higher SC likelihood. The square boxes in Figure 4C indicate the contacts that transit from negative to positive, before and after applying this rule. Although more false-positive errors are newly introduced than the false negative errors are eliminated, they are intended as the same strategy discussed above, which makes less false-negative errors. Overall, the PR curve for varying SVM threshold is shifted to the right when this rule is applied (Figure 4E).

### VC-Net Architecture and Training

In a naive approach, the synapse candidates found by the procedure so far could be considered as the final result of synapse prediction. The SC-Net would implicitly exploit the same visual cues, including the VC, as human experts do, because SC-Net utilizes the context information in the large field of a view. However, to further improve the accuracy and to assign the pre- and postsynaptic neurons, we introduced the VC-Net to explicitly utilize the visual cues of the VCs.

The VC-Net takes a volume of image as its input and produces the likelihood map of the voxels belonging to a VC as the output. The architecture of the VC-Net is almost identical to the SC-Net except for a few parameters and the fact that the VC-Net does not have dropout layers (Supplementary Figure 2B). The same data augmentation strategy was used as the case of the SC-Net. The loss function is the sigmoid cross-entropy error. The learning rate was kept at 1. × 10^–3^ throughout the training for fast convergence. The training was terminated after 1.62 million updates (Supplementary Figure 3B).

### Synapse Prediction and Assignment Using Vesicle-Cloud Likelihood

The output of the VC-Net is the map of voxel-wise likelihood that a voxel is in a VC (Figure 5A). The segments of individual VCs were obtained from the map by the similar method used for the segmentation of neurons, which uses a watershed algorithm to aggregate the similar voxels (Turaga et al., 2010; Zlateski and Seung, 2015). The VC likelihood was considered to represent the affinity between neighbor voxels (Turaga et al., 2010). The voxel-wise likelihood map was converted to a 3D-undirected affinity graph by repeating the likelihood values to three axes. To prevent a VC from hanging across multiple neurons, the likelihood map was masked by the neuronal boundaries. A watershed algorithm was used to turn the affinity map into segmentation (Figure 5B; Zlateski and Seung, 2015). The parameters for watershed were selected from many experiments (data not shown).

The VC segmentation is used to predict the synapses from the synapse candidates. For each synapse candidate, the distance to the closest VC is measured where the VC needs to be inside either of the pair of neuronal segments forming the contact. The distance is defined as the voxel distance between the closest VC voxel and the contact voxel of the synapse candidate (Figure 5C). If the distance is 5 or less, the synapse candidate is considered to have a corresponding VC, and it is finally predicted as a synapse. At this stage, the SVM threshold −0.2 selected above yields the highest F1-score, 0.955 (Figure 5D). We also tried the VC size threshold as the parameter, because too small VCs might be noise and need to be discarded. The result shows that the VC size threshold 1,000 voxels yields the highest F1-score (Figure 5D), and the parameter is accepted. Lastly, the neuron to which the VC belongs is assigned as the presynaptic neuron. The other one naturally becomes the postsynaptic neuron. The synapse type is determined based on the type information and excitatory vs. inhibitory nature of the presynaptic neuronal fragment.

### Structure and Connectivity Analysis

All the analyses were performed by custom-written MATLAB codes. The size of the synapse was calculated from the contact size as follows. As the anisotropic volume has a voxel size 12 nm × 12 nm × 50 nm, a face of the contacting voxel has the area 600 nm^2^ in the YZ or ZX plane and 144 nm^2^ in the XY plane. The size of the contact can be calculated by counting the contacting faces for each axis direction. The number of contacting faces can be counted during the contact extraction step. It is the same as the number of locations that the segment ID changes along the XYZ axes.

The number of synapses per bouton was found as follows. The VC was considered to be unique to a bouton, and a VC was used as the proxy of a bouton. During the last step of the synapse detection, a VC was matched for each synapse. Here, we counted the number of synapses that were matched to a VC.

## Results

### Reconstruction and Synapses in the Test Set

The results are discussed and evaluated for the test set. The volume of the test set (11.9 μm × 11.9 μm × 12.4 μm) is 0.15% of the entire data. It contains 598 neuronal fragments in total, which consist of 574 PFs, 4 PCs, 17 INs, and 3 CFs (Figure 3A). Two neuronal fragments (1 glial cell and 1 Golgi cell) were not reconstructed nor considered in this work. All the reconstructed neuronal fragments were neuropils, except one IN soma. About 57.6% of voxels of the volume belong to reconstructed neuronal fragments, and the chief proportion of the remaining voxels belong to glial cells. Other proportion includes small numbers of PFs and INs. Since the brain sample was sectioned and imaged roughly sagittally, the PFs align parallelly and the PCs align perpendicularly with the Z axis (Eccles et al., 1967; Kim and Augustine, 2021). All the PFs pass through both sides of the volume along the Z axis. The PCs are roughly laminated, each occupying one lamina. The CFs and INs appear to irregularly arborize at this scale.

The 598 neuronal segments yield a total of 5,857 contacts, 1,973 of which are the relevant contacts (Table 3). The majority of the relevant contacts involve the PFs. About 59.5% are PF-PC (*n* = 1,173) and 36.8% are PF-IN (*n* = 726). The remaining 74 relevant contacts consist of 44 IN-PC, 13 IN-IN, and 17 CF-PC.

**TABLE 3:** Accuracy for the test set by cell type.

Pre-Post (cell count)	Relevant Contacts (%)	Total Synapses (%)	Non-synapses (%)	Ambiguous (%)	FP	FN	TP	Precision	Recall	F1-score
PF (489) – PC (4)	1,173 (59.5)	360 (70.9)	675 (58.3)	138 (45)	9	11	349	0.975	0.969	0.972
PF (370) – IN (17)	726 (36.8)	116 (22.8)	451 (38.6)	159 (51.8)	14	9	107	0.884	0.922	0.903
CF (3) – PC (4)	17 (0.9)	8 (1.6)	7 (0.6)	2 (0.7)	0	0	8	1	1	1
IN (14) – PC (4)	44 (2.2)	21 (4.1)	20 (1.7)	3 (1)	0	3	18	1	0.857	0.923
IN (6) – IN (4)	13 (0.7)	3 (0.6)	5 (0.4)	5 (1.6)	0	0	3	1	1	1
Total (598)	1,973	508	1,158	307	23	23	485	0.955	0.955	0.955

*The synapse detection accuracies for different cell-type pairs are given together with the basic metrics.*

**FIGURE 6:**
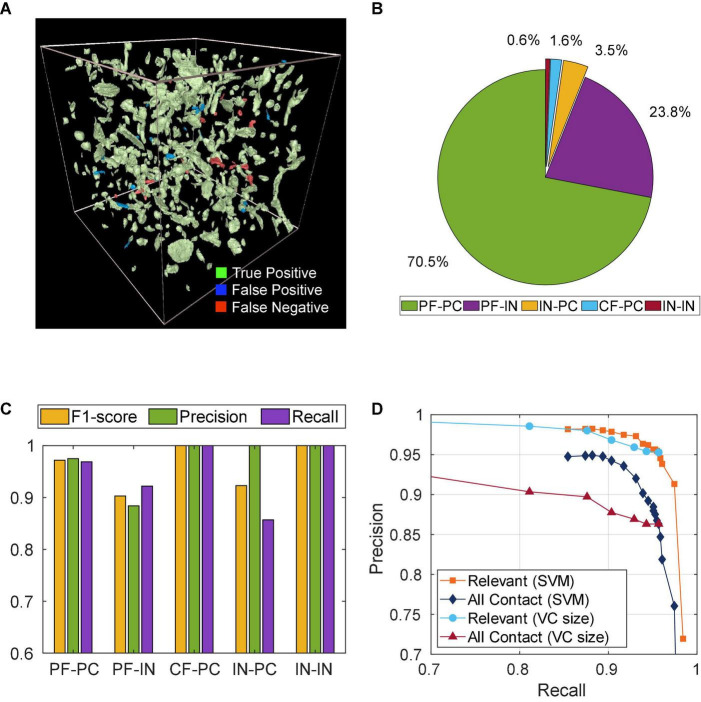
Visualization of the accuracy. **(A)** The result of synapse classification for the test set is displayed in 3D mesh rendering. The contacts are color coded as true positive (green), false positive (blue), or false negative (red). **(B)** The proportion of the synapses by the types is shown in a pie graph. The PF-related synapses are absolutely dominant. **(C)** The accuracy measures by the types are shown in bar graphs. The PF-IN is the least accurate (90.3% F1-score) followed by IN-PC (92.3%) and then by PF-PC (97.2%). **(D)** The impact of the cell-type restriction step to select the relevant contacts is tested. The PR curves are shifted to down-left when all the contacts are used (the highest F1-score, 92.6%) compared to when the relevant contacts are used (95.5%), for both varying SVM thresholds and varying VC size thresholds. The cyan curve for relevant contacts for varying VC size thresholds is identical here and, in Figure 5D, serves as a reference.

From these, 508 are labeled as synapses in the ground truth (Figure 6A). About 1,158 and 307 are labeled as non-synaptic and ambiguous, respectively. About 70.9% of the total synapses are between PF and PC (*n* = 360), and 22.8% are between PF and IN (*n* = 116). Of the remaining 32 synapses, 21 are IN-PC, 3 are IN-IN, and 8 are CF-PC (Figure 6B and Table 3).

### Accuracy Evaluation

The final accuracy of the proposed method was measured to be 0.955 in F1-score (Figures 5D, 6C,D). We estimated the impact of each contact selection rule on the accuracy. We calculated the F1-score when the contacts selected by the rules are assumed to be the final prediction for synapses. The highest F1-score for varying SVM thresholds, when the voxel-fraction-based SVM rule is applied to the relevant contacts, is 0.923. After the voxel-count-based rule (NHSCLV) is applied, the highest F1-score increases to 0.934. The use of VC further raises the F1-score to the final value, 0.955. The utilization of the VCs in addition to the SC likelihood raised the accuracy beyond the 0.95 barrier (Table 1).

The details of accuracy can be evaluated for different contact types, which are determined by the pairs of neuronal-fragment types (Figure 6C and Table 3). The PFs seem to be the major source of errors as they are involved in the majority of the contacts. The PF-IN is least accurate (90.3%) followed by IN-PC (92.3%) and then by PF-PC (97.2%). The accuracies of other contact types appear high, but the number of instances is too small to conclude. Further investigation shows that the errors are most common when the size of the synapse is small in all contact types. They tend to have thin and vague PSD, a small contact area, and a small VC. The low resolution of the image further aggravates the accurate decision for small synapses (data not shown). The PF-related errors occur mainly because the PF synapses are inherently small. The IN synapses have varied sizes, and most of the IN-involved errors occur for small synapses (section “Synapse Size and Density”). A few error examples are shown in Supplementary Figures 5, 6.

Next, we wondered about the impact of type restriction and using relevant contacts (Figure 6D). When the type restriction was not applied and all the contacts were used instead of the relevant contacts, the PR curves for varying SVM thresholds and for varying VC size thresholds are shifted toward down-left, compared to the cases when the relevant contacts were used. The maximum F1-score when the relevant contacts are not used is 0.926 as opposed to the final F1-score, 0.955. While the 92.6% of accuracy is still very competitive to those in the pieces of literature, it is clear that the type restriction enhanced the accuracy even more.

The contacts that were labeled as ambiguous in the ground truth were excluded from the accuracy calculation. We estimated the lower bounds of the accuracy when they were included. If all the ambiguous contacts were actually synapses, then our prediction yields 0.832 F1-score. If all the ambiguous contacts were, indeed, non-synapses, the F1-score of our prediction became 0.862. These values seem to be too low compared to the highest value of 0.955; however, they are still competitive to a few other methods (Table 1). The exclusion of ambiguity is advantageous for the training of AI and is often adopted for accuracy evaluation as well (Dorkenwald et al., 2017).

### Synapse Size and Density

In the following sections, we demonstrate the usability of the proposed method by investigating the properties of synapses and connectivity between CML neurons. First, we measured the size of the synapses. The size of a synapse was calculated from the number of voxels in the synaptic contact (see section “Structure and Connectivity Analysis”).

**FIGURE 7:**
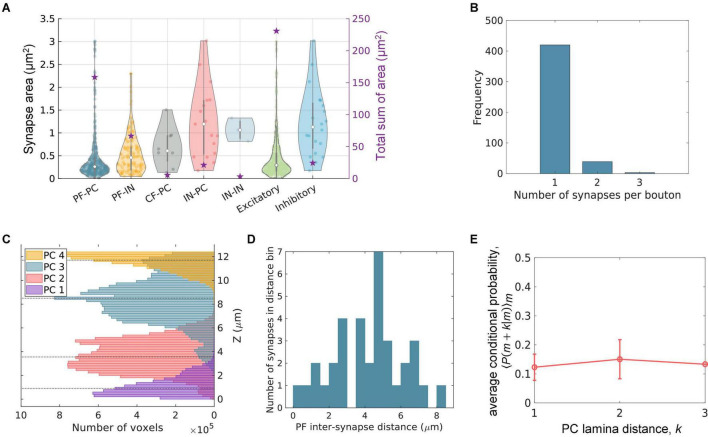
Structural properties of the synapses and connectivity. **(A)** The size of the synapses measured by the contact area is shown grouped by the cell types or by excitatory (PF-PC, PF-IN, CF-PC) or inhibitory (IN-PC, IN-IN) types (left y-axis). The violin plots roughly show the distribution. The empty circles are the median, and the vertical gray bars range from the 25th to 75th percentiles. The total sum of the synapse area of each type is denoted by the star (right y-axis). **(B)** Frequency of the boutons with 1∼3 synapses. The boutons with 2 or more synapses are 9% (42 out of 462). **(C)** The volume distribution of the 4 PCs along the Z axis in the test set was calculated. The PCs have laminar organization, where each PC occupies one lamina. The dashed lines are the median for each distribution. The distance between adjacent PCs is estimated to be 4.5 ∼ 5.5 μm, considering the fact that PCs 1 and 4 are cut off by the borders of the volume. **(D)** The histogram of inter-synapse intervals of PF-PC synapses. A peak near 5 μm is coincident with the inter-PC distance in panel **(C)**. **(E)** The mean conditional probability that a PF connects also to PC (*m + k*), given that it connects to PC (*m*), averaged over *m*. The constant conditional probability implies that there is no correlation between the PF connections to different PCs. The error bars are standard error.

The synapse size was estimated for each contact type (Figure 7A, left four columns). The median area of PF-PC synapses (0.26 μm^2^) was much smaller than that of IN-PC synapses (1.2 μm^2^). The PF-IN (0.46 μm^2^) and CF-PC synapses (0.61 μm^2^) have a small median synaptic area, too. This is because the excitatory neurons (PF and CF) tend to innervate the dendritic spines, and inhibitory neurons (IN) tend to innervate dendritic shafts as is well known (Eccles et al., 1967). This is clearer when the five contact types are grouped into excitatory and inhibitory types (Figure 7A, right to columns). The median area of the excitatory synapses (0.29 μm^2^) was much smaller than that of the inhibitory synapses (1.13 μm^2^).

The size of PF synapses has the smallest average, median, and variation at the same time, but there are many outliers whose size is larger than the median or average by several folds. The size of the outliers was overestimated by the size of the entire contact. The contacts of PFs are often formed in an elongated shape (Figure 6A), only a part of which is the synaptically relevant contact corresponding to the PSD. We visually inspected the biggest outliers, and all of them were the case.

Such overestimation can be quantitatively estimated using the ground truth labels (see section “Ground Truth and Data Labeling”). The fraction of the size change before and after the erasion of synaptically irrelevant parts of the contacts exhibits a skewed distribution. The 25% of the contacts did not change in size. The median of the fraction of size change is 20%. Considering the 20% of change as typical, the median of the synaptically relevant area of the contacts is estimated to be 0.24 μm^2^. This value is larger than the calculation from the images of fluorescently labeled postsynaptic proteins, 0.12 ∼ 0.13 μm^2^ (Zhu et al., 2018). Since this difference can potentially undermine rigorous analyses, we plan to improve the method to manage this issue.

The spatial density of the synapses for different contact types is 0.005/μm^3^ (CF-PC), 0.002/μm^3^ (IN-IN), 0.01/μm^3^ (IN-PC), 0.07/μm^3^ (PF-IN), and 0.203/μm^3^ (PF-PC). The PF-PC synapses outnumber all the rest synapses by far. These densities are underestimated because the reconstruction is not complete. When the size of each synapse and the density of the synapses are considered together, the total sum of the area of PF-PC synapses (158.59 μm^2^) is much larger than that of IN-PC synapses (21.27 μm^2^). The excitatory neurons jointly provide a larger total synaptic area (230.86 μm^2^) than the inhibitory neurons do altogether (24.48 μm^2^).

### Multiple-Synaptic Boutons

One presynaptic bouton can innervate multiple postsynaptic sites, where the multiple sites can be either on one neuron or on multiple neurons. We inspected the number of postsynaptic sites that one presynaptic bouton innervates (Figure 7B). The boutons that make more than one synapse were found to be 9%, 42 out of the total number of boutons, 462. This number is probably underestimated, because the reconstruction is not complete. The multiple-synaptic boutons are known to have various functional roles including those related to synaptic plasticity (Harris, 1995; Kim et al., 2019).

### Laminar Organization of the Cerebellar Molecular Layer

The dendrites of a PC form a flat arborization along the sagittal plane and different PC dendrites align parallelly to one another. The PFs are parallel to one another and perpendicular to the PC arborization (Eccles et al., 1967). This laminar organization of the CML was quantified for the test set.

We calculated the volume distribution of the PCs by counting the number of voxels along the Z axis (Figure 7C). The distances between adjacent PCs can be determined from the median of each distribution. The maximum distance 5 μm is measured between the two PCs at the center. The average distance is 3.6 μm; however, it is an underestimation because the PCs on both sides of the Z axis are cut off by the border of the data. Therefore, the typical inter-PC distance is assumed to range between 4.5 and 5.5 μm.

We then measured the distance between adjacent PF-PC synapses on a PF along the Z axis. The location of a synapse is represented by the median voxel of the contact. The PF-PC inter-synapse distances are broadly distributed with a prominent peak near 4.5 ∼ 5 μm (Figure 7D). The typical PF-PC inter-synapse distance is consistent with the inter-PC distance.

Lastly, we tested the correlation of PF-PC connections (Figure 7E). Here, we considered cell-to-cell connectivity rather than the synaptic level connections. Let us call the four PCs as PC 1∼4, in order of increasing Z. We measured the fraction of the PFs that synapse also to PC (*m* + *k*) among the PFs that synapse to PC (*m*). It is a conditional probability that a PF has a connection to PC (*m* + *k*), given the condition that it has a connection to PC (*m*). We computed the mean of the conditional probability over *m*. It appears that the average conditional probability is roughly constant in the small test set. The result suggests that a PF makes a connection to a PC independently of whether or not it has a connection to another PC in a close distance.

## Discussion

We report an accurate and automated synapse detection method for cerebellar EM connectomics. It exhibits over 95% of accuracy with full automation. It provides the location, type, size, and direction of the synapses without human intervention. The over 95% accuracy was accomplished for the first time for any data (Table 1). The result is remarkable, considering that the accuracy for the cerebellar sample is lower than those for other brain regions by the same method in a previous report because the cerebellar synapses are small and dense (Becker et al., 2013).

The high accuracy of this method can be attributed to a few factors. First, it utilizes two deep learning AIs, the SC-Net and the VC-Net, which were trained with large amount of data. The VC-Net complements the SC-Net, while they exploit the same visual cues that human experts refer to. Second, the parameters such as the SVM threshold and the VC size threshold were carefully fine-tuned for the test data, and they may be close to the optimum for the entire data we will analyze. Third, the idea of relevant contact and type restriction greatly increased the accuracy. It is particularly beneficial for the case of PFs. The PFs are tightly packed forming bundles and make many contacts with each other (Figure 3C). They may result in many false positive errors without the exclusion of the irrelevant PF-PF contacts (Supplementary Figure 6).

The method has a few limitations, too. First, the reconstruction is a prerequisite (Staffler et al., 2017; Parag et al., 2018; Buhmann et al., 2021). Other methods are needed if a researcher wants to reconstruct only a few neurons first, find their synapses, and then backward trace the synaptic partners of the first neurons from the synapses. Second, the method regards an entire contact as the synapse even when the PSD is only at a small part of it (Staffler et al., 2017). This needs to be improved in the future studies. Third, the type restriction by relevant contacts may limit the chance for the exploration of unknown connections. However, the method can still be used to test whether there exist unknown connections for given types, only if the contacts of those types are set to be relevant. In such cases, however, the accuracy may be low.

Most of the synapse detection software in the pieces of literature, including this work, consists of many files of source code rather than a readily executable program. It is a hard task to reproduce the reported results even for the researchers with expert-level computational skills. There had been a few executable software packages, which were claimed to be easily usable, to be generally applicable, and to have good accuracy (Morales et al., 2011; Becker et al., 2013). Nevertheless, they have been recently replaced by newer and more accurate technologies equipped with deep learning AIs. The AIs have to be trained and fine-tuned before application. It is unfortunate particularly for the common neurobiologists with basic computational skills. Efforts are being made toward generally usable software with AI (Staffler et al., 2017; Buhmann et al., 2021).

The proposed method can easily scale up as the computation time for processing the test set is less than 20 min on a desktop computer with 8 CPU cores and 32 GB memory. While the method is tweaked for, and may seem to be limited to, the cerebellum and the data, the approaches and ideas may be extended to other brain regions as well. The requirements for the extension can include the change in the network architecture of the AIs, new training of the AIs with the image data from the region, fine tuning of the parameters, and the region’s having well-defined cell types and type connectivity. Nevertheless, the more important lesson of this study may be is the idea that new specialized designs exploiting tissue-specific properties of the different brain regions will enhance the performance of the methods. For example, there can be different policies to replace the type restriction for other brain regions. The high accuracy of the method for individual synapses is advantageous for the inspection of subcellular wiring specificity. The connectomic analyses on the small test set already showed interesting results. We plan to report the larger-scale analyses of the entire data soon. All in all, the method is practically useful for large-scale cerebellar connectomics.

## Data Availability Statement

The raw data supporting the conclusions of this article will be made available by the authors, without undue reservation.

## Ethics Statement

The animal study was reviewed and approved by KBRI Committee for Animal Research.

## Author Contributions

JK designed the study with inputs from CP and JG. CP and JG wrote the codes and analyzed the data with the supervision of JK. KL advised on biology. SL advised on computation. CP and JK interpreted the data and wrote the manuscript with inputs from JG, SL, and KL. All authors contributed to the article and approved the submitted version.

## Conflict of Interest

The authors declare that the research was conducted in the absence of any commercial or financial relationships that could be construed as a potential conflict of interest.

## Publisher’s Note

All claims expressed in this article are solely those of the authors and do not necessarily represent those of their affiliated organizations, or those of the publisher, the editors and the reviewers. Any product that may be evaluated in this article, or claim that may be made by its manufacturer, is not guaranteed or endorsed by the publisher.
