# Supplementary material for: Automated Synapse Detection Method for Cerebellar Connectomics
Source: Front Neuroanat. 2022 Mar 11;16:760279. doi: 10.3389/fnana.2022.760279 (PMC8963724; doi:10.3389/fnana.2022.760279)
Supplement: Supplementary file 1 [file Data_Sheet_1.PDF]

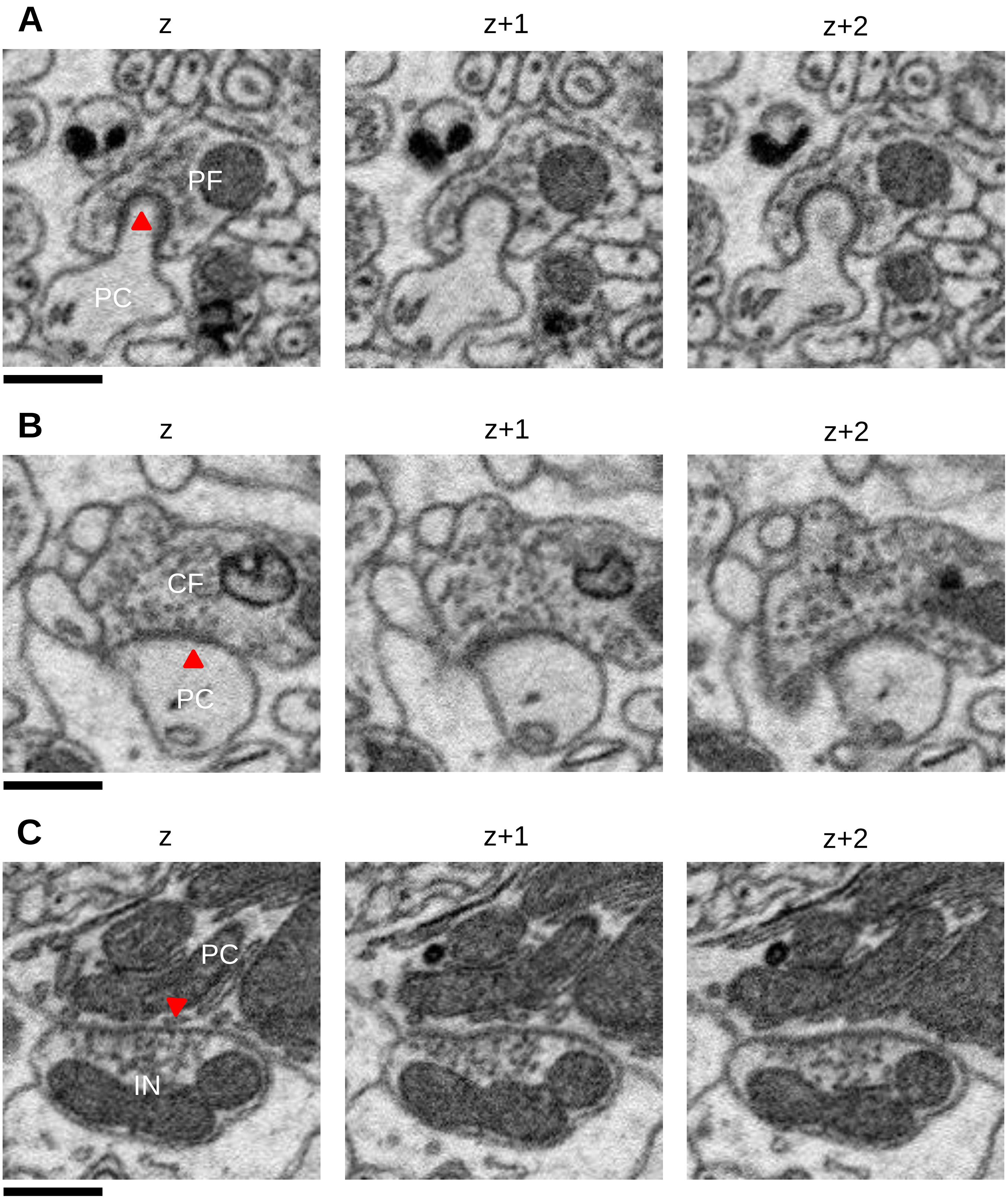

Supplementary Figure 1: Examples of synapses between various cell types. 3 sets of 3 consecutive sections show **(A)** an excitatory synapse between a PF bouton and a PC spine, **(B)** an excitatory synapse between a CF bouton and a PC spine, and **(C)** an inhibitory synapse between an IN bouton and a PC dendritic shaft, respectively. Human experts and the proposed method find the synapses by referring to the visual cues of the vesicles and PSD. The cell types were separately identified by the morphologies of the cells in 3D representation of large-scale reconstruction. Scale bars: 600 nm, 50 voxels.

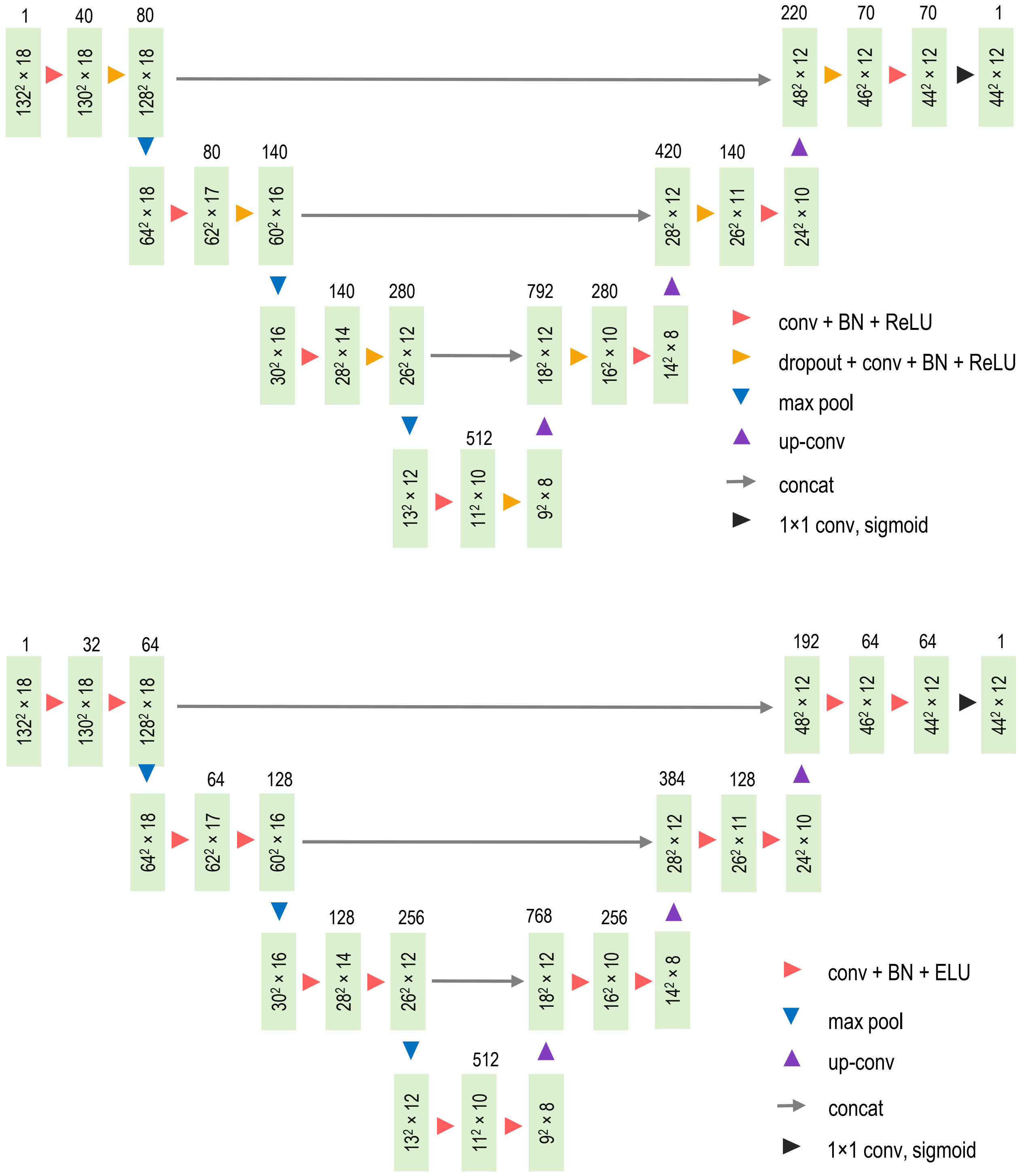

Supplementary Figure 2: Network architectures of the SC-Net (top) and the VC-Net (bottom). The architecture is adopted from the 3D U-Net and a few parameters, such as the size of the filters, were modified.

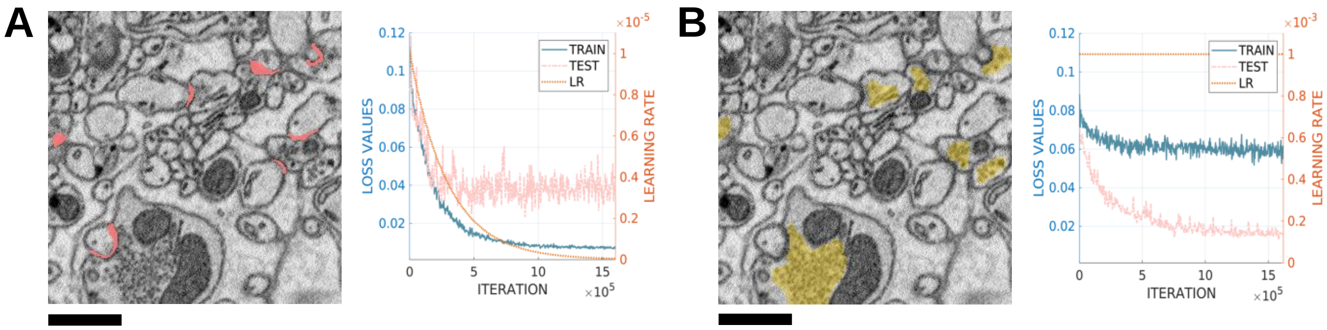

Supplementary Figure 3. Examples of the ground truth and learning curves during training. **(A)** The synaptic contacts are labeled in red voxels as the ground truth (left). The SC-Net learning curves for the training set and the test set show decaying errors (loss values) as the training progresses. The learning rate (LR) is being lowered (right). **(B)** Same as **A** for the VC-Net. The LR is kept constant. Scale bars: 900nm, 75 voxels.

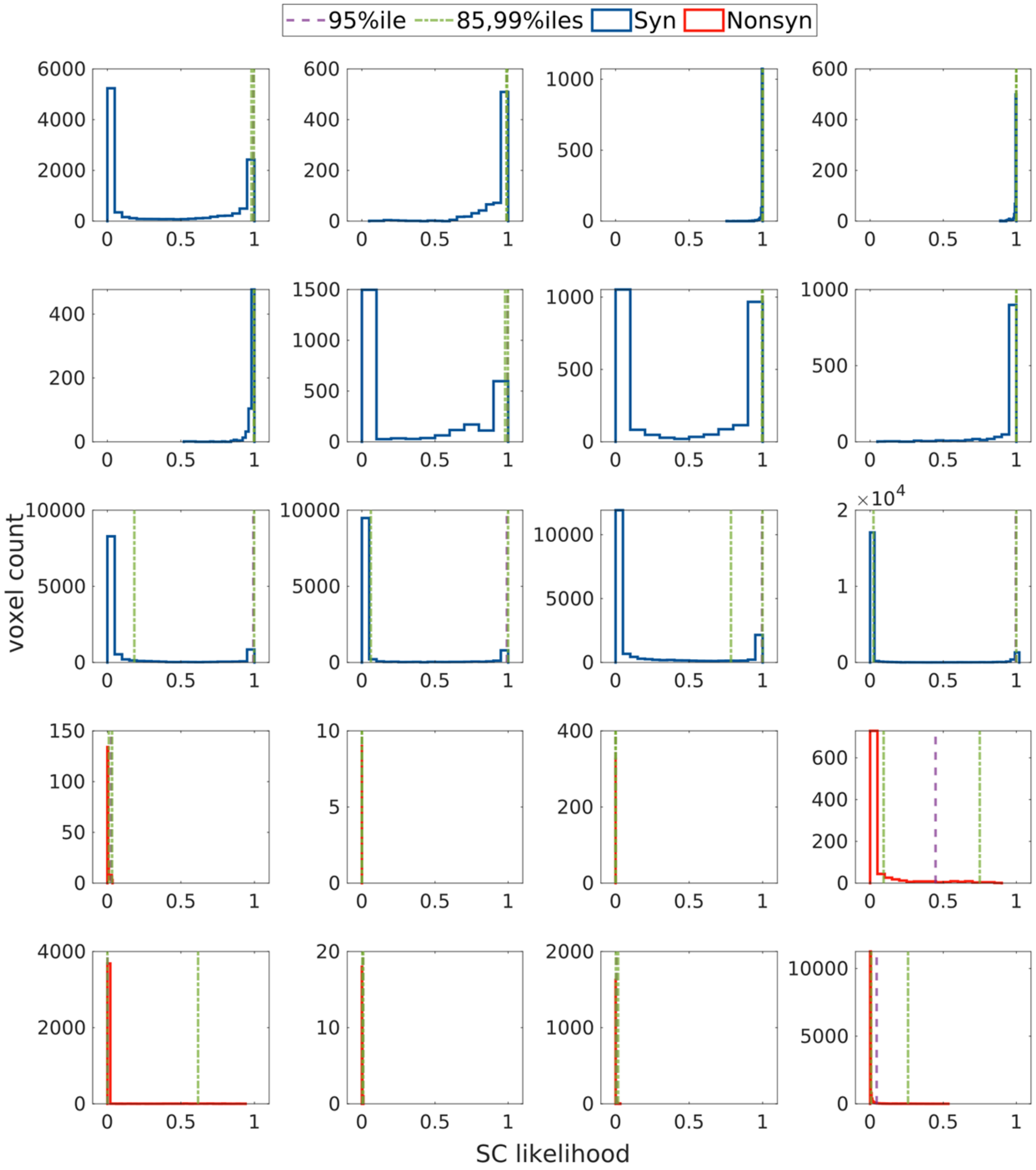

Supplementary Figure 4. SC-likelihood distributions for selected contacts. The SC-likelihood distributions for synaptic contacts (blue, first three rows) and for non-synaptic contacts (red, last two rows) are shown, together with the three percentiles in vertical lines. The SVM which uses the percentile measures, correctly classifies the first two rows as synaptic and the last two rows as non-synaptic, but it misclassifies the third row as non-synaptic while they are synaptic contacts. It is because the SC-likelihood distributions in the third row have high peaks on the left while the SVM assumes peaks on the right for synaptic contacts. The peaks appear on the left for the synaptic contacts in the third row because the contacts are larger than synaptic active zones.

A

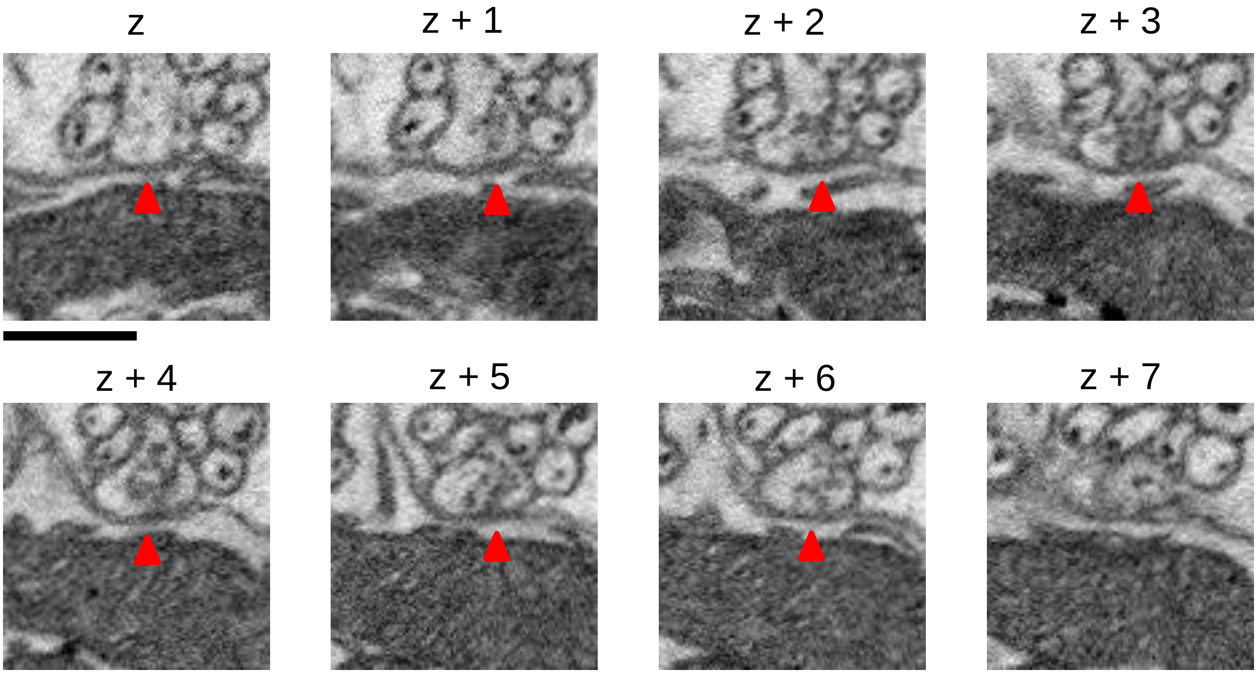

B

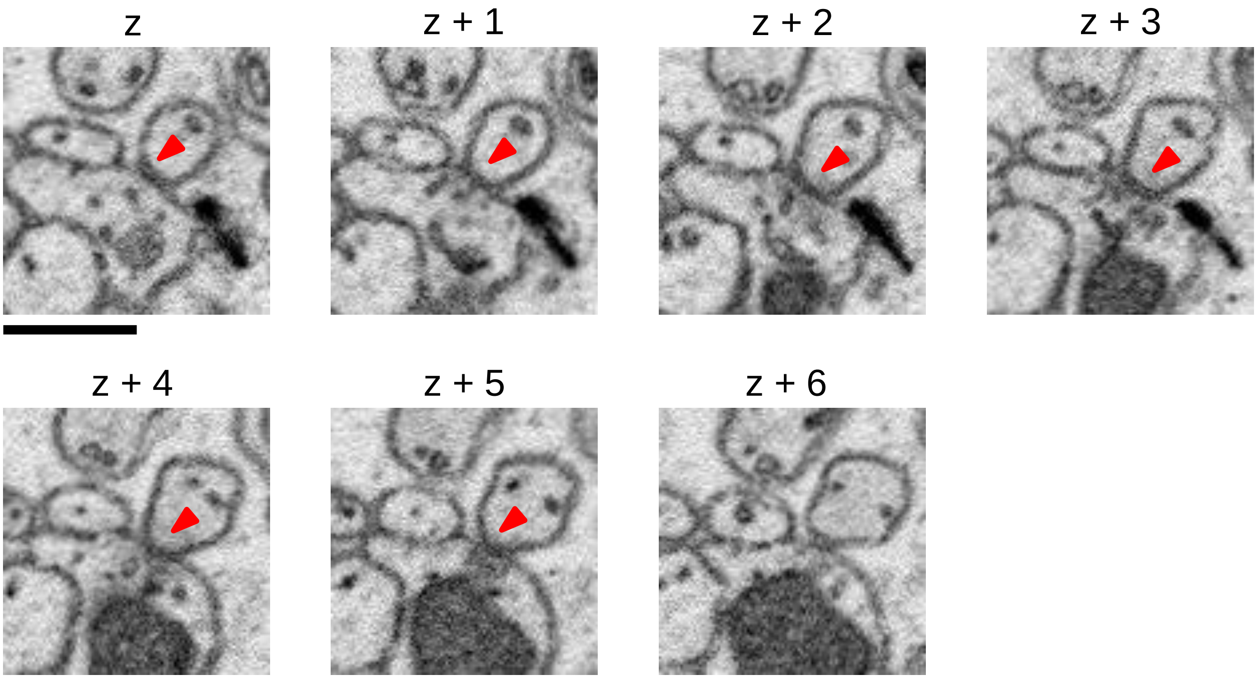

C

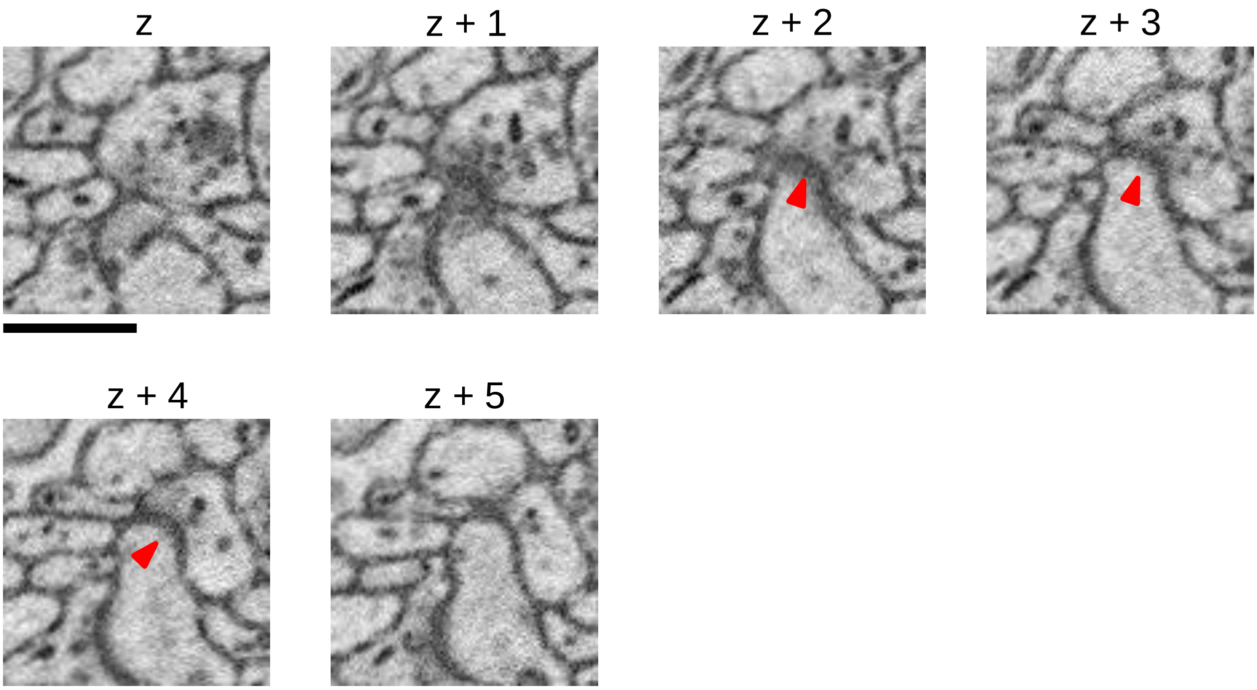

Supplementary Figure 5: False negative error examples. (A) Consecutive slices for an inhibitory synapse between an IN axon and PC dendritic shaft. **(B)** and **(C)** Two sets of consecutive slices for two excitatory synapses, each of which are between a PF bouton and a PC spine. The false negative synapses are indicated by red arrows. Scale bars: 600nm, 50 voxels.

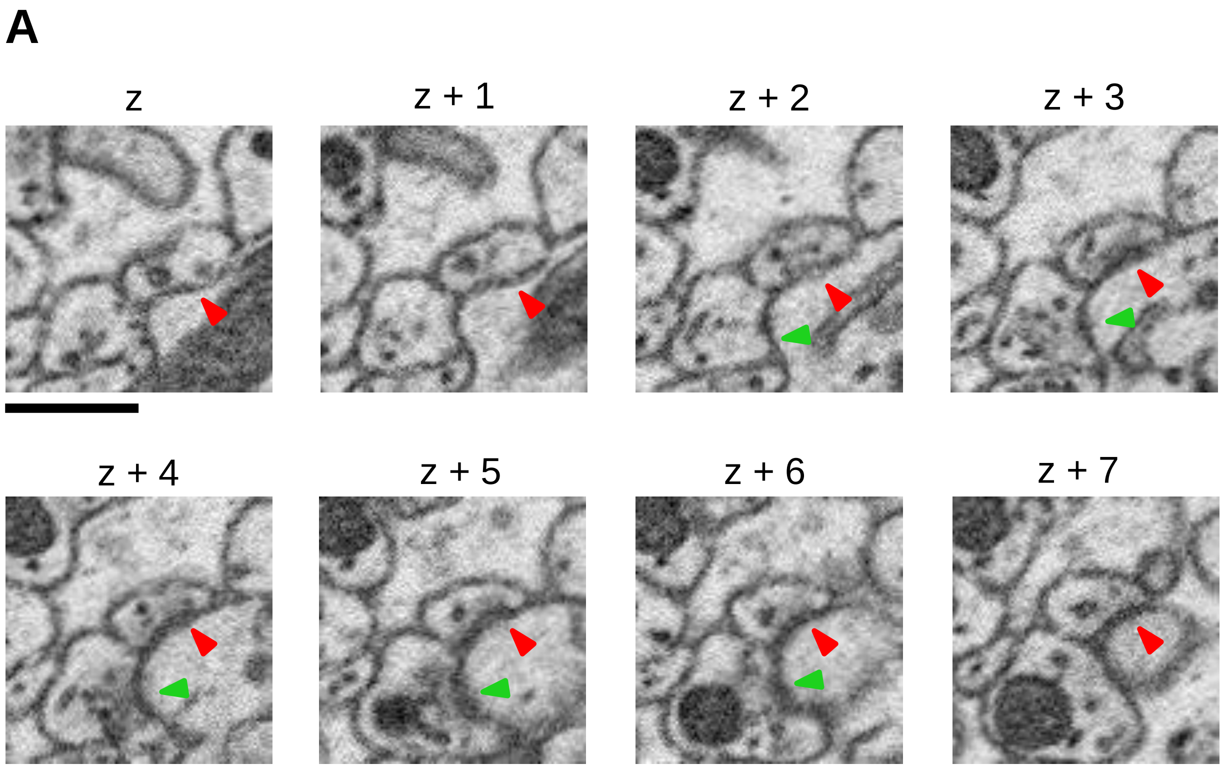
A
z
z + 1
z + 2
z + 3
z + 4
z + 5
z + 6
z + 7

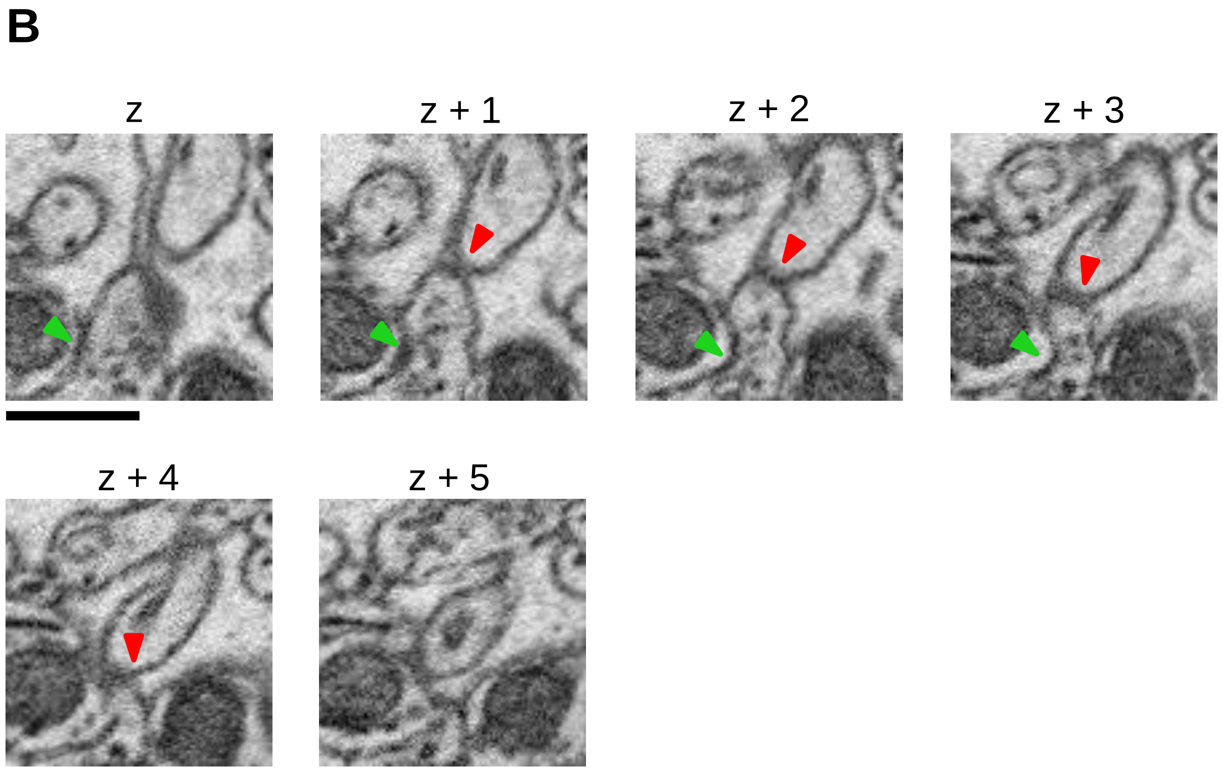
B
z
z + 1
z + 2
z + 3
z + 4
z + 5

C

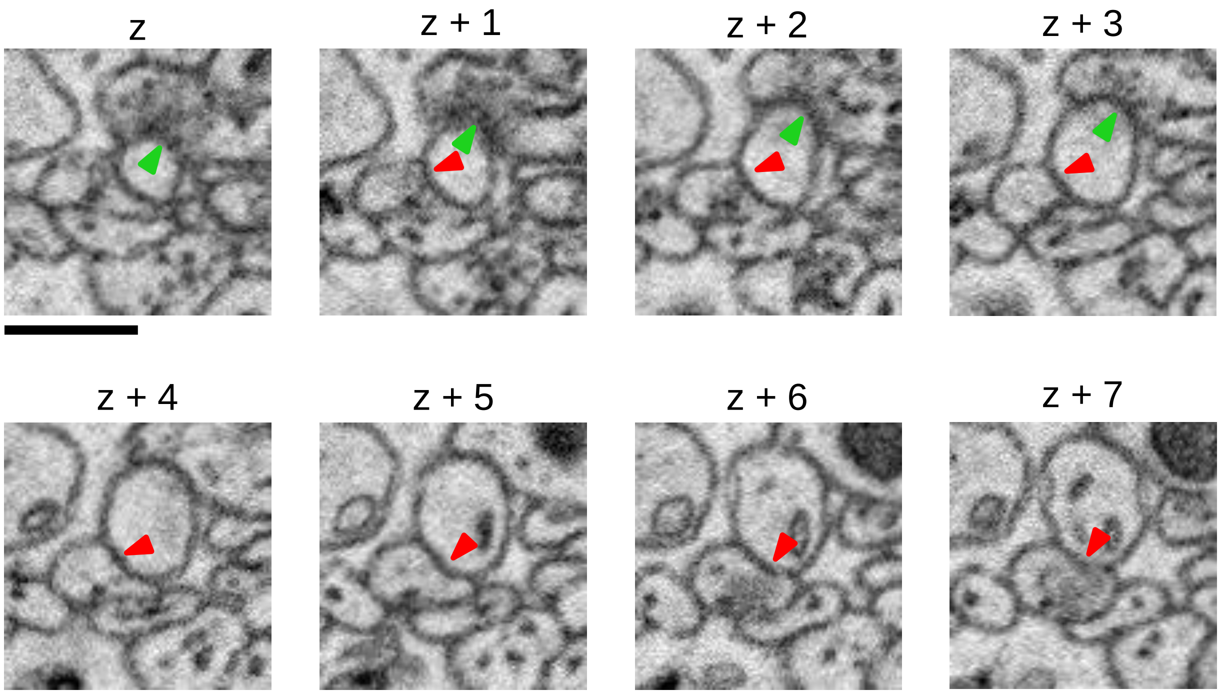

Supplementary Figure 6. False positive error examples. **(A)** Consecutive slices for a false positive error (indicated with red arrows) between a PF and an IN. A true positive synapse is in a close distance (indicated with green arrows). **(B)** Consecutive slices for a false positive error between a PF and a PC spine. A synaptic contact and a vesicle cloud are in a close distance. **(C)** Consecutive slices for a false positive error between a PF and a PC spine. A small structure in the PF resembling a vesicle cloud is confusing. A true positive PF to PC spine synapse is on the opposite side. Scale bars: 600 nm, 50 voxels
